# The Impact of Maternal Graves’ Disease on Neonatal Thyroid Function: A Systematic Review

**DOI:** 10.7759/cureus.75041

**Published:** 2024-12-03

**Authors:** Maria Tzoraki, Grigorios Karampas, Antigoni Sarantaki, Aikaterini Lykeridou, Christina Kanaka-Gantenbein, Dimitra Metallinou

**Affiliations:** 1 Medical Service Department, Hygeia Hospital, Athens, GRC; 2 Second Department of Obstetrics and Gynaecology, Aretaieio University Hospital, Medical School, National and Kapodistrian University of Athens, Athens, GRC; 3 Department of Midwifery, School of Health and Care Sciences, University of West Attica, Athens, GRC; 4 Division of Endocrinology, Diabetes and Metabolism, 1st Department of Pediatrics, "Aghia Sophia" Children's Hospital, Medical School, National and Kapodistrian University of Athens, Athens, GRC

**Keywords:** maternal graves’ disease, maternal hyperthyroidism, neonatal hyperthyroidism, neonatal hypothyroidism, thyroid function

## Abstract

Maternal Graves' disease (GD) poses a significant risk to neonatal thyroid function due to the transplacental transfer of thyrotropin receptor antibodies (TRAbs). This systematic review aims to assess the impact of maternal GD on neonatal thyroid outcomes and identify key maternal factors influencing these outcomes. A comprehensive literature search was conducted across PubMed, Scopus, and Cochrane, resulting in the inclusion of 18 studies published from 2014 to 2024. The review focused on neonates born to mothers with active or previous GD and investigated the effects of various maternal treatments, including antithyroid drugs (ATDs), radioactive iodine (RAI) therapy, and thyroidectomy on their offspring. The findings indicate that elevated maternal TRAb levels are a strong predictor of neonatal thyroid dysfunction, with neonates exhibiting conditions such as hyperthyroidism, transient thyrotoxicosis, or hypothyroidism. The incidence of neonatal thyroid dysfunction ranged from 0.1% to 5% in pregnancies complicated by GD, with higher rates observed in cases requiring long-term ATD therapy. Neonatal outcomes varied, with some cases resolving after appropriate treatment, while others necessitated prolonged monitoring due to risks of developmental delays and complications. The review highlights the importance of early third-trimester TRAb screening and regular neonatal thyroid function testing within the first week of life. Although neonatal outcomes were generally favorable with prompt diagnosis and treatment, the review emphasizes the need for standardized protocols to optimize monitoring and management strategies in pregnancies complicated by GD. Further research should explore long-term neurodevelopmental outcomes and evaluate the impact of different maternal treatment strategies on neonatal thyroid health.

## Introduction and background

Maternal hyperthyroidism, particularly in the context of Graves' disease (GD), poses a significant threat to neonatal thyroid function. GD is an autoimmune disorder characterized by the presence of thyroid-stimulating immunoglobulins (TSI), a type of thyrotropin receptor antibody (TRAb), which can cross the placenta and influence the fetal thyroid gland [1]. This can result in a variety of neonatal thyroid disorders, including hyperthyroidism, transient hyperthyroidism, congenital hypothyroidism, transient hypothyroidism and central hypothyroidism, each carrying distinct clinical challenges [2].

The incidence of neonatal thyroid dysfunction among infants born to mothers with Graves' disease (GD) ranges from 0.1% to 2.7% [3]. TRAbs, in particular, play a critical role, as they can either stimulate or inhibit thyroid function, resulting in neonatal hyperthyroidism or hypothyroidism, respectively. Elevated maternal TRAb levels (≥2.5 IU/L) and neonatal TRAb levels (≥6.8 IU/L) are strong indicators of neonatal thyroid dysfunction [4], which typically emerges between 18 and 20 weeks of gestation when the fetal thyroid begins to express thyroid-stimulating hormone (TSH) receptors [5]. Neonatal hyperthyroidism (NH) is a rare condition, affecting between 1% and 5% of newborns whose mothers have had either active or previous GD. Approximately one in 50,000 newborns is affected, an incidence four times higher than that of transient neonatal hypothyroidism caused by maternal TSH receptor-blocking antibodies. Some mothers carry both stimulating and blocking antibodies, and the balance between these antibodies can fluctuate over time, affecting the severity and type of thyroid dysfunction [6]. Furthermore, maternal treatment with antithyroid drugs (ATDs) can introduce additional complications in the neonate, as these medications cross the placenta and may cause fetal or neonatal hypothyroidism. High doses of ATDs are linked to neonatal hypothyroidism [7], complicating the management of such pregnancies.

The available literature on neonatal thyroid outcomes in the context of maternal GD is fragmented. Variability in reported study designs and differing maternal treatment regimens contribute to this inconsistency, making it difficult to develop standardized management protocols for at-risk neonates. Neonatal thyroid dysfunction, particularly if undiagnosed or inadequately managed, can result in long-term developmental issues, including cognitive impairment, growth delays, and metabolic disturbances [5]. With hypothyroidism being the most common preventable cause of intellectual disability in children and neonatal hyperthyroidism potentially leading to serious complications such as heart failure and impaired growth [1], the findings of a systematic review would have significant implications for improving neonatal health outcomes. In addition, it would help clinicians to better understand the role of maternal factors and antithyroid drug use, providing clearer guidance on managing hyperthyroidism in pregnancy.

This systematic review seeks to address the impact of maternal GD on neonatal thyroid function, and how maternal factors, such as TRAb levels, antithyroid drug usage, and maternal thyroid control affect neonatal thyroid outcomes. The purpose of the study is to provide an overview of the current state of research, identify gaps in the existing literature and synthesize the available evidence to guide clinical practice for managing pregnancies complicated by maternal GD. By consolidating data, this review aims to develop a clearer understanding of neonatal thyroid outcomes, provide insights into optimal monitoring and management protocols for at-risk neonates, influence future policy-making and direct research efforts to tackle the gaps highlighted in the literature.

## Review

Materials and methods

Study Design

The present systematic review was conducted in line with the Preferred Reporting Items for Systematic Reviews and Meta-Analyses (PRISMA) guidelines [8]. The PICO (Population, Intervention, Comparison, Outcome) model was employed to formulate the objectives of the review. The patient population (P) comprises all neonates born to mothers with GD. The intervention (I) comprises the maternal thyroid management during pregnancy (e.g. ATD use, radioactive iodine therapy (RAI), thyroidectomy, TSI, TRAb concentration monitoring). Comparison (C) is to neonates born to mothers without GD or without other thyroid dysfunction during pregnancy. The primary outcome (O) to be evaluated is the neonatal thyroid function.

Eligibility Criteria

The review included cohort studies, case-control studies, and clinical trials examining neonatal thyroid function abnormalities in relation to maternal GD. Studies that involved neonates born to mothers diagnosed with GD were included, regardless of whether maternal GD was treated or untreated, or managed with ATDs, RAI or thyroidectomy. Case reports, reviews, editorials, letters, book chapters and animal studies were excluded. Studies published before 2014 and in other languages than English were also excluded.

Literature Search

A comprehensive literature search was performed across PubMed, Scopus, and the Cochrane Central Register of Controlled Trials (CENTRAL) from 2014 up until October 2024. The search strategy included combinations of the following keywords and medical subject heading (MeSH) terms: “neonate”, “infant”, "Graves' disease", "neonatal thyrotoxicosis", "neonatal hypothyroidism", "transplacental antibodies", "TRAb", "thyroid-stimulating immunoglobulins", "antithyroid drugs", and "neonatal thyroid dysfunction." Boolean operators "AND" and "OR" were used to combine keywords and ensure inclusivity. The search strategy was adapted for each database. A manual search of reference lists from included studies was also conducted to identify additional relevant articles.

Study Selection

**Figure 1:**
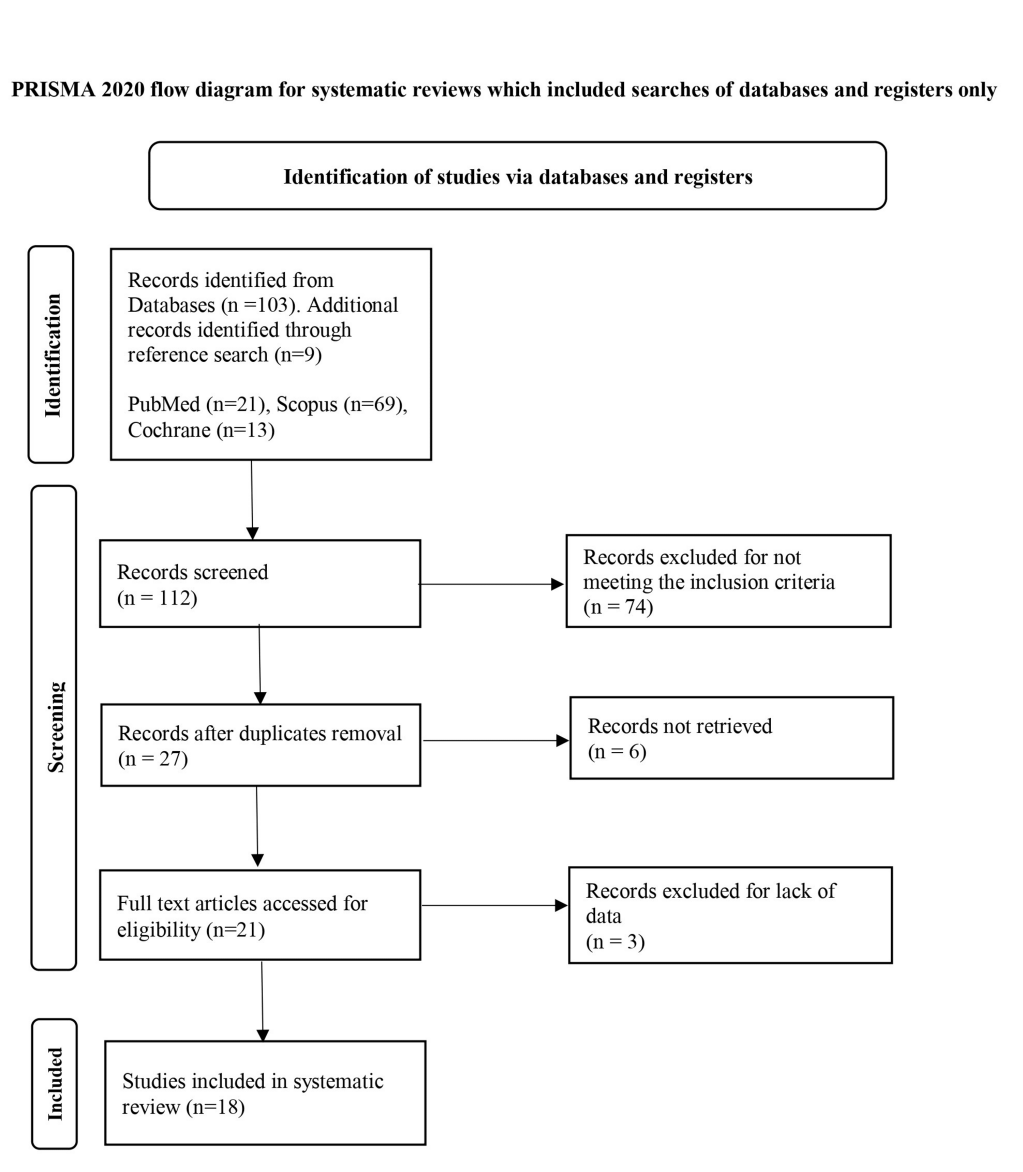
PRISMA 2020 flow diagram for new systematic reviews PRISMA: Preferred Reporting Items for Systematic Reviews and Meta-Analyses; n: number

The study selection process followed PRISMA guidelines [8] (Figure 1). Two independent reviewers screened titles and abstracts for relevance. Full-text articles were reviewed for eligibility based on predefined inclusion and exclusion criteria. Discrepancies between reviewers were resolved through discussion and consensus.

**Figure 2:**
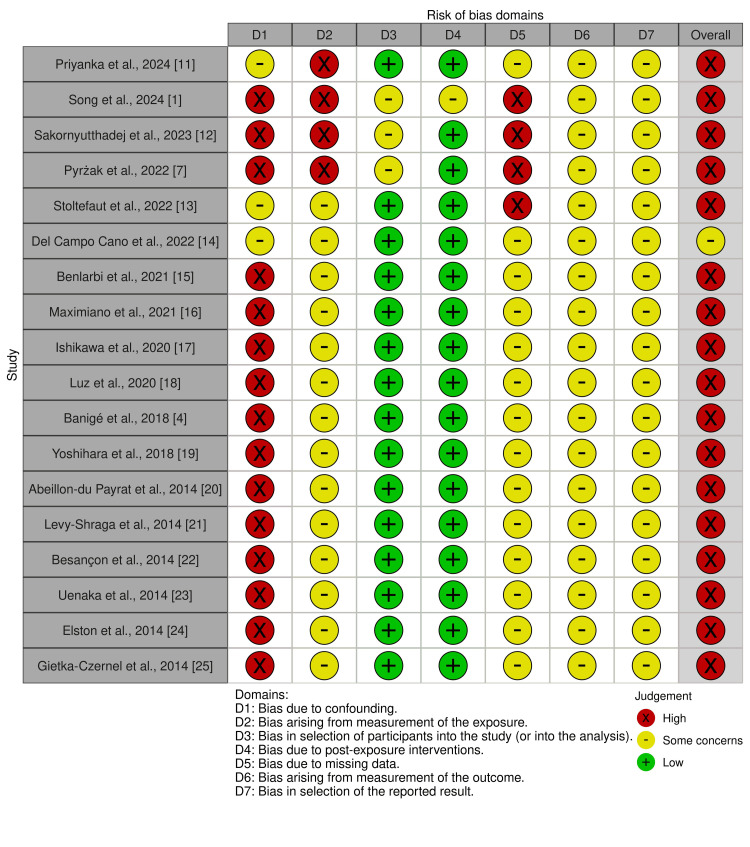
Risk of bias assessment using the ROBINS-E tool: "Traffic light" plot demonstrating the overall risk of bias in included studies. Sources: Refs. [1, 4, 7, 11-25] D: domain; ROBINS-E: Risk of Bias in Non-Randomized Studies of Exposure

All 18 studies were assessed using the ROBINS-E (Risk of Bias in Non-Randomized Studies of Exposure) tool [9]. All studies demonstrated a high risk of bias, except for one. The evaluation covered potential biases across seven domains and the results are visualized using a “traffic light” plot (Figure 2) to show domain-level judgments for each study [10]. This visualization highlighted key areas of concern with most issues related to potential confounding, measurement of the exposure and missing data. These results emphasize the typical challenges faced in observational research, especially in retrospective studies.

Data Extraction and Synthesis

Data were extracted by two independent reviewers using a standardized data extraction form. Extracted data included study characteristics (sample size, maternal thyroid status, neonatal outcomes), treatment details (ATDs, RAI, thyroidectomy, TRAb levels), and neonatal thyroid dysfunction (hypothyroidism, thyrotoxicosis, and long-term outcomes). Data were synthesized using a qualitative approach, focusing on the types of neonatal thyroid dysfunction reported and their associated maternal factors.

Results

This systematic review includes 18 studies investigating the effects of maternal GD on neonatal thyroid function. The sample sizes across these studies ranged from 13 to 415 neonates, with a total of over 1506 cases. The majority of studies followed a retrospective cohort design, examining the neonatal thyroid outcomes in mothers treated for GD either before or during pregnancy. Maternal treatments varied, with most mothers receiving ATDs, RAI, surgery, or levothyroxine (LT4) (Table 1).

**Table 1:** Characteristics of studies involved in this systematic review. Sources: Refs. [1, 4, 7, 11-25] GD: Grave’s disease; N: number; ATD: antithyroid drugs; CBZ: carbimazole; mg/d: milligrams per day; T2: second trimester; T3: third trimester; RAI: radioiodine; LT4: levothyroxine; T1: first trimester; TD: thyroidectomy; PTU: propylthiouracil; MMI: methimazole; N/M: not mentioned; KI: potassium iodine

Author and Year [Reference]	Study Type	Study sample [Neonates] (N)	Maternal Treatment (ATD/Radioiodine/Surgery)	Neonatal Hyperthyroidism (Transient/Thyrotoxicosis/GD)(N)	Neonatal Hypothyroidism (Central/Primary/Transient)(N)	Prematurity (N)	Death due to thyroid dysfunction (N)
Priyanka et al., 2024 [11]	Retrospective Cohort	51	Pre-pregnancy diagnosis: 32 women; ATD (CBZ median dose: 15mg/d T2 to 5 mg/d T3) Diagnosis during pregnancy: 19 women; ATD (CBZ, median dose: 25 mg/d T2 to 10 mg/d T3)	7/0/0	2/4/4	5	5
Song et al., 2024 [1]	Retrospective Cohort	15	Pre-pregnancy diagnosis: 6 women; 3 women ATD, 2 women RAI+LT4, 1 woman ATD+LT4. Diagnosis during pregnancy: T1: 8 women ATD, T3: 1 woman ATD	15/0/0	0/0/0	8	0
Sakornyutthadej et al., 2023 [12]	Retrospective Cohort	262	Timing of diagnosis is not specified. 253 women (7 of them had 2 pregnancies and 2 of them had twins.) Pre-pregnancy treatment: 56 women mentioned; 22 women TD, 34 women RAI; of them, 53 women on LT4 During pregnancy treatment: [trimester is not mentioned], 19 women: ATD, 1 woman PTU+MMI, 4 women MMI (+KI+ examethasone in some: number not specified), 118 pregnancies: ATD, 118 pregnancies disease remission: 76 pregnancies treatment not mentioned, 42 mothers LT4	0/0/12	0/5/7	46	0
Pyrżak et al., 2022 [7]	Retrospective Cohort	13	Pre-pregnancy diagnosis and treatment: 7 women; 3 women ATD, 2 women RAI+LT4, 1 woman TD+LT4, 1 woman no treatment. During pregnancy treatment: 2 women LT4, 1 woman PTU, 1 woman MMI+Encorton, 1 woman LT4+MMI, 1 woman PTU+LT4, 1 woman MMI diagnosis during pregnancy: T1: 6 women; 1 woman MMI, 2 women PTU to MMI, 2 women no treatment, 1 woman MMI to LT4	5/6/0	0/0/0	2	0
Stoltefaut et al., 2022 [13]	Retrospective Cohort	42	Pre-pregnancy diagnosis: 42 women; 17 women LT4, 5 women CBZ, 3 women MMI, 4 women PTU, 13 women no treatment. Diagnosis during pregnancy: 0 women.	0/0/0	0/0/0	0	0
Del Campo Cano et al., 2022 [14]	Prospective Cohort	33	Pre-pregnancy diagnosis: 32 women; 6 women TD, 7 women RAI, 19 women ATD. During pregnancy treatment: 5 women ATD. Diagnosis during pregnancy: 1 woman	0/0/0	0/0/0	1	0
Benlarbi et al., 2021 [15]	Prospective Cohort	32	Pre-pregnancy diagnosis: 21 women: 4 women TD+LT4, 1 woman RAI+LT4, 1 woman TD+RAI+LT4, 12 women ATD, 1 woman CBZ, 1 woman LT4, 1 woman not specified. During pregnancy treatment: 1 woman no treatment, 14 women PTU, 6 women LT4. Diagnosis during pregnancy: 11 women; 4 women CBZ, 7 women PTU.	23/0/0	3/2/0	9	0
Maximiano et al., 2021 [16]	Retrospective Cohort	31	30 women. Pre-pregnancy diagnosis: 27 women; treatment: not mentioned. During pregnancy treatment: 16 pregnancies, no treatment; T1: 6 pregnancies MMI, 4 pregnancies PTU, 5 pregnancies LT4; T2: 3 pregnancies MMI, 7 pregnancies PTU, 5 pregnancies LT4; T3: 6 pregnancies MMI, 4 pregnancies PTU, 5 pregnancies LT4. Diagnosis during pregnancy: T1: 1 woman, T2: 1 woman, T3: 1 woman.	2/0/0	0/0/0	N/M	0
Ishikawa et al., 2020 [17]	Prospective Cohort	80	Pre-pregnancy diagnosis: 80 women; 5 women TD, 4 women RAI, 31 women ATD, 40 women: N/M. During pregnancy treatment: 44 women no treatment, 4 women MMI, 22 women PTU, 17 women LT4. Diagnosis during pregnancy: 0 women.	12/0/0	0/0/7	N/M	0
Luz et al., 2020 [18]	Retrospective Cohort	50	Pre-pregnancy diagnosis: 46 women (48 pregnancies, 2 cases of multiple pregnancies); 1 woman TD, 16 women RAI, 29 women N/M. During pregnancy treatment: T1: 10 women MMI, 15 women PTU, 11 women LT4; T2: 10 women MMI, 13 women PTU, 14 women LT4; T3: 9 women MMI, 11 women PTU, 15 women LT4. Diagnosis during pregnancy: 0 women.	2/0/0	0/0/3	2	0
Banigé et al., 2018 [4]	Retrospective Cohort	415	Pre-pregnancy diagnosis: 415 women; 175 women LT4, 145 women ATD, 95 women N/M. During pregnancy treatment: not specified. Diagnosis during pregnancy: 0 women.	23/0/0	0/0/0	0	0
Yoshihara et al., 2018 [19]	Retrospective Cohort	145	Pre-pregnancy diagnosis: 145 women; 145 women RAI 2 years before pregnancy. During pregnancy treatment: T1: 54 women on ATD or KI, 91 women: N/M. Diagnosis during pregnancy: 0 women.	8/0/0	0/0/0	N/M	0
Abeillon-du Payrat et al., 2014 [20]	Retrospective Cohort	47	Pre-pregnancy diagnosis: 45 women (2 twin pregnancies); 7 women RAI+LT4, 7 women TD+LT4, 14 women ATD, 3 women ATD+LT4, 1 woman no treatment, 1 woman lost to follow-up, 10 women ATD stopped during pregnancy because of remission of GD, 2 women N/M. Diagnosis during pregnancy: 0 women.	9/0/0	0/0/3	N/M	0
Levy-Shraga et al., 2014 [21]	Retrospective Cohort	96	Pre-pregnancy diagnosis: 96 women. Treatment pre-pregnancy: N/M. During pregnancy treatment: 43 women PTU, 3 women MMI, 16 women no treatment, 15 women LT4, 19 women N/M. Diagnosis during pregnancy: 0 women.	83/0/4	0/0/0	10	0
Besançon et al., 2014 [22]	Retrospective Cohort	68	Pre-pregnancy diagnosis: Not specified. During pregnancy treatment: 27 women no ATD, 41 women ATD. Diagnosis during pregnancy: Not specified.	7/0/0	0/0/0	10	1
Uenaka et al., 2014 [23]	Retrospective Cohort	35	Pre-pregnancy diagnosis: 32 women (1 twin pregnancy), treatment: ATD. Diagnosis during pregnancy: T2/3: 2 women, treatment: N/M.	5/0/0	1/0/3	7	4
Elston et al., 2014 [24]	Retrospective Cohort	49	Pre-pregnancy diagnosis: 29 women; 12 women TD (22 pregnancies: 11 women on LT4), 17 women RAI (27 pregnancies; 15 pregnant women on LT4, 5 pregnant women on ATD, 7 pregnant women no treatment). Diagnosis during pregnancy: 0 women.	0/1/0	0/0/0	0	8
Gietka-Czernel et al., 2014 [25]	Prospective Cohort	42	The timing of the diagnosis is not specified. Past GD: 16 women; 10 women RAI + LT4, 4 women TD+LT4, 1 woman LT4, 1 woman not specified treatment. Current GD: 26 women; 16 women ATD: (10 women stop ATD during T2/ T3, 6 women ATD dosing adjustments), 3 women RAI, 7 women N/M. 1 woman TD during pregnancy + LT4.	2/0/0	0/0/2	N/M	0

Maternal TRAb levels were reported in most studies, and a clear association was found between elevated maternal TRAb levels and neonatal thyroid dysfunction. Priyanka et al. [11] and Pyrżak et al. [7] highlighted that higher maternal TRAb levels increased the likelihood of neonatal hyperthyroidism or transient thyrotoxicosis, while Sakornyutthadej et al. [12] also found a correlation between elevated maternal TRAb and the occurrence of neonatal Graves’ disease (NGD), particularly in neonates whose mothers had the highest TRAb levels. Maximiano et al. [16] reported two cases of neonatal hyperthyroidism and highlighted the importance of timely testing and TRAb titers to prevent neonatal thyroid dysfunction. Similarly, Abeillon-du Payrat et al. [20] demonstrated that maternal thyrotropin-binding inhibitory immunoglobulins (TBII) value over 5 IU/L (international units/liter) was indicative of neonatal hyperthyroidism risk, further supporting the predictive value of TRAbs. Gietka-Czernel et al. [25] highlighted that monitoring fetal thyroid health with ultrasound (US), especially when combined with maternal TRAb measurements, is valuable for an early detection and management of fetal thyroid disorders.

In contrast, Stoltefaut et al. [13] and Del Campo Cano et al. [14] did not observe significant correlations between TRAb levels and neonatal clinical outcomes, suggesting that elevated TRAb levels may not always directly translate into predictable clinical effects.

Priyanka et al. [11] noted that seven of 46 live neonates developed transient neonatal thyrotoxicosis. Song et al. [1] reported that 13 out of 15 neonates exhibited overt hyperthyroidism, though not all required treatment. Sakornyutthadej et al. [12] diagnosed NGD in 12 neonates who required antithyroid drugs and Levy-Shraga et al. [21] in a cohort of 96 neonates reported 83 cases of hyperthyroidism and four of NGD. In the study by Benlarbi et al. [15], neonates born to mothers treated with RAI developed both hyperthyroidism and central hypothyroidism post-treatment. Additionally, central hypothyroidism was documented in total six cases across different studies [11, 15, 23], all of which identified uncontrolled maternal GD as the main cause. Most studies confirmed these trends, with neonates often presenting with thyroid dysfunction early in life, though many showed signs of resolution following appropriate treatment or monitoring.

Long-term outcomes were generally favorable, although some cases required prolonged follow-up, especially when neonatal TRAb levels were high. Although rare, neonatal mortality was reported in the study by Priyanka et al. [11], where five out of 51 neonates died, possibly due to complications from maternal hyperthyroidism. Other common complications included fetal goiter, low birth weight, and preterm delivery. Ishikawa et al. [17] identified hemodynamic changes in neonates with higher TRAb-positive mothers, noting elevated levels of N-terminal pro B-type natriuretic peptide (NT-proBNP) and free thyroxine compared to TRAb-negative neonates. Developmental delay was reported in a few cases. For example, Song et al. [1] documented a case of developmental delay following methimazole (MMI) treatment, and Sakornyutthadej et al. [12] identified developmental issues in some neonates diagnosed with NGD.

Additionally, Elston et al. [24] found that maternal treatment type influenced TRAb levels, with mothers who underwent surgery more likely to have negative TRAb levels compared to those treated with RAI, suggesting differences in neonatal outcomes based on treatment modality. Some studies [12, 16, 17, 24, 25], reported discrepancies in the data regarding maternal treatment, possibly due to cases involving multiple pregnancies or variations in maternal treatment during pregnancy that were not consistently reported. These variations could contribute to differences in neonatal thyroid health, underscoring the importance of recording data in detail when considering maternal treatment factors to interpret neonatal outcomes.

Discussion

This systematic review aimed to evaluate the impact of maternal GD on neonatal thyroid function by synthesizing data from studies conducted in countries such as Spain, Japan, Poland, France, Portugal, China, Thailand, and New Zealand. These countries represent diverse iodine statuses, with some showing adequate iodine levels, while others have either insufficient or excessive iodine intake. This variation in iodine intake is particularly relevant in the context of fetal thyroid status in mothers with Graves' disease, as iodine levels can influence both maternal thyroid function and the efficacy of treatments such as ATDs, further impacting the fetal thyroid [26]. We reviewed 18 studies, encompassing 1506 neonates born to mothers diagnosed with GD. Untreated thyroid dysfunction in neonates can lead to significant long-term health complications [27]. While individual studies have explored this association, our review offers a comprehensive and up-to-date synthesis of evidence, highlighting the variability in neonatal outcomes based on maternal treatments and TRAb levels. This systematic review is original in its scope, providing a detailed analysis of maternal treatment regimens (e.g., ATDs; RAI; surgery), their effects on neonatal thyroid function, and insights into optimal monitoring and management strategies for affected neonates. By integrating data from recent studies, we aimed at filling existing gaps in knowledge and emphasizing the importance of tailored maternal care to mitigate neonatal risks.

The evidence consistently shows that the implications of GD extend beyond the mother, significantly impacting the thyroid health of her neonate. The primary mechanism by which maternal GD affects neonatal thyroid function is the transplacental transfer of thyroid-stimulating antibodies (TSI/TRAb) [18]. These antibodies can stimulate or, less commonly, block the fetal thyroid gland, leading to conditions ranging from hyperthyroidism to hypothyroidism. TSIs cross the placenta, particularly after the fetal thyroid becomes functional around the 12th week of gestation [12]. Understanding these mechanisms is crucial for anticipating and managing neonatal thyroid disorders.

Neonatal thyrotoxicosis occurs in 1%-5% of neonates born to mothers with GD and is characterized by symptoms such as tachycardia, irritability, poor weight gain, and in severe cases, heart failure and craniosynostosis [6]. These findings are supported by Banigé et al. [4] and Levy Shraga et al. [21], who underscore the need for prompt diagnosis and treatment to prevent long-term neurodevelopmental impairments. However, as noted by Priyanka et al. [11], neonatal thyrotoxicosis can be delayed if maternal ATDs were administered during pregnancy, as these drugs cross the placenta and temporarily suppress the fetal thyroid. Neonatal hyperthyroidism is typically treated with beta-blockers such as propranolol and methimazole to manage symptoms and reduce thyroid hormone production [28]. Severe cases may require more aggressive therapies, including systemic corticosteroids or iodine and a prolonged stay in the neonatal intensive care unit [4].

In contrast, maternal ATD therapy can suppress fetal thyroid function, leading to neonatal hypothyroidism [29]. Primary neonatal hypothyroidism may also result from maternal TRAb blocking the TSH receptor [7]. Symptoms such as lethargy, feeding difficulties, hypotonia, and delayed bone age are indicative of hypothyroidism, which, if not promptly treated, can lead to permanent neurocognitive deficits. As reported by Abeillon-du Payrat et al. [20], delays in treatment due to unrecognized hypothyroidism can have serious consequences, emphasizing the need for early screening and intervention.

Though rare, central hypothyroidism can occur in the context of maternal GD, particularly when excessive maternal thyroxine (T4) suppresses the neonatal hypothalamic-pituitary-thyroid axis [15]. Unlike primary hypothyroidism, central hypothyroidism is characterized by low or inappropriately normal TSH levels with low T4. Case studies confirm this condition [11, 23]. Neonatal hypothyroidism, though often transient as maternal antibodies wane, requires immediate LT4 therapy to support normal brain development [30].

The guidelines recommend monitoring maternal TRAb levels in the third trimester as an essential step in assessing the risk of neonatal thyroid dysfunction [26]. Elevated maternal TRAb levels in the third trimester strongly predict neonatal hyperthyroidism, as reported in previous studies [4, 7]. Although maternal TRAb and ATD requirements typically decrease during late pregnancy, paradoxical increases can occur, particularly in severe or newly diagnosed cases, or in the presence of other comorbidities such as diabetes or hypertension [11]. The importance of using the lowest effective dose of ATDs, such as methimazole or propylthiouracil, is emphasized to maintain maternal thyroid levels within a trimester-specific target range [31]. This approach helps minimize the risk of both maternal hyperthyroidism and fetal hypothyroidism. Close monitoring of TRAb levels and adjustment of maternal treatment regimens are essential to mitigate risks to the neonate.

Furthermore, regular neonatal screening for thyroid dysfunction within the first week of life is crucial, particularly in high-risk neonates, born to mothers with high TRAb levels or those treated with ATDs during pregnancy. Diagnosing neonatal thyroid dysfunction involves measuring free T4 and TSH levels, and in some cases, TRAb testing. As noted by Uenaka et al. [23], close follow-up is essential because thyroid status can evolve, with neonates initially appearing euthyroid but developing symptoms later.

Although some studies, such as that by Del Campo Cano [14], found no direct correlation between maternal TRAb levels and neonatal outcomes, the overall evidence supports a strong association between maternal and neonatal thyroid function, particularly in neonates born to mothers with poorly controlled GD or paradoxical increases in TRAb during pregnancy. Fetal ultrasound findings, including goiter and tachycardia, as reported by Czernel et al. [25], provide early indicators of fetal thyroid dysfunction and should be part of routine monitoring for at-risk pregnancies.

The variability in neonatal outcomes can often be traced to inconsistencies in TRAb testing and follow-up protocols. For instance, Weissenfels et al. [32] found that only one out of seven neonates from mothers with GD was tested for TRAb, despite recommendations for screening neonates at risk of hyperthyroidism [26]. Such inconsistencies highlight the critical need for standardized protocols that ensure all neonates born to mothers with GD receive appropriate testing and follow-up.

Our review underscores the importance of early and precise monitoring of both maternal and neonatal thyroid function. Measuring maternal TRAb levels in the third trimester is essential for assessing the risk of neonatal hyperthyroidism. Based on our findings, we recommend a protocol that includes regular neonatal screening for thyroid dysfunction within the first week of life, especially in infants born to mothers with high TRAb levels or those treated with ATDs during pregnancy. Neonates diagnosed with thyroid dysfunction should be managed in collaboration with pediatric endocrinologists to ensure appropriate treatment and monitoring of long-term outcomes.

Strengths and Limitations of the Review

A major strength of this review is its comprehensive analysis of studies published after 2014, incorporating both prospective and retrospective data. Additionally, we focused exclusively on primary research, excluding case reports, reviews, and animal studies, providing a more robust synthesis of available evidence. This approach enhances the relevance of our findings to clinical practice, offering insight into neonatal thyroid dysfunction linked to maternal GD.

Despite these strengths, there are several limitations. Many of the included studies had small sample sizes, which may limit the generalizability of the findings. Furthermore, variability in maternal treatment protocols, study designs, and follow-up durations contributed to heterogeneity, making it challenging to perform a formal meta-analysis. Additionally, there was limited long-term follow-up in many studies, leaving gaps in understanding the prolonged impacts of neonatal thyroid dysfunction.

Recommendations for Future Research

Future research should aim to explore the long-term neurodevelopmental outcomes of neonates with thyroid dysfunction related to maternal GD. Comparative studies evaluating different maternal treatment strategies (e.g., ATDs vs. surgery) and their impact on the neonatal thyroid are needed. Additionally, studies assessing the role of maternal comorbidities, such as diabetes and hypertension, on neonatal thyroid outcomes would provide valuable insights into more personalized management strategies for pregnant women with GD.

## Conclusions

Neonates born to mothers with GD are at significant risk for thyroid dysfunction due to the transplacental passage of maternal TRAb, TSIs, and ATDs. Thyroid dysfunction in these neonates can range from thyrotoxicosis to hypothyroidism, with the potential for significant clinical consequences if not promptly recognized and managed. Early detection through neonatal screening and appropriate treatment are essential to ensuring optimal neurodevelopmental outcomes. Ongoing monitoring of thyroid function in these neonates is necessary, as thyroid status can evolve in the early months of life due to changing maternal antibody levels and the metabolism of medications.

Given the potential for adverse neonatal outcomes stemming from maternal hyperthyroidism, systematic monitoring of thyroid function in newborns of hyperthyroid mothers is essential. Early detection and intervention for thyroid disorders can significantly improve health outcomes. The importance of establishing comprehensive clinical guidelines and standardized protocols for managing these pregnancies and postnatal follow-up according to the different populations, cannot be overstated, as timely management can prevent the long-term complications associated with untreated thyroid dysfunction.
